# Ethnicity and Cutaneous Melanoma in the City of Sao Paulo, Brazil: A Case-Control Study

**DOI:** 10.1371/journal.pone.0036348

**Published:** 2012-04-27

**Authors:** Olinda C. Luiz, Reinaldo José Gianini, Fernanda T. Gonçalves, Guilherme Francisco, Cyro Festa-Neto, José Antonio Sanches, Gilka J. F. Gattas, Roger Chammas, José Eluf-Neto

**Affiliations:** 1 Laboratório de Epidemiologia e Imunobiologia – LIM38, Departamento de Medicina Preventiva da Faculdade de Medicina da Universidade de São Paulo, Sao Paulo, Brazil; 2 Laboratório de Processamento de Dados Biomédicos – LIM39, Departamento de Medicina Preventiva da Faculdade de Medicina da Universidade de São Paulo, Sao Paulo, Brazil; 3 Pontifícia Universidade Católica de São Paulo, Sorocaba, Brazil; 4 Laboratório de Imuno-hematologia e Hematologia Forense – LIM40, Departamento de Medicina Legal, Ética Médica, Medicina Social e do Trabalho, Faculdade de Medicina da Universidade de São Paulo, Sao Paulo, Brazil; 5 Laboratório de Oncologia Experimental – LIM24, Departamento de Radiologia, Faculdade de Medicina da Universidade de São Paulo, Sao Paulo, Brazil; 6 Departamento de Dermatologia, Faculdade de Medicina da Universidade de São Paulo, Sao Paulo, Brazil; Centers for Disease Control and Prevention, United States of America

## Abstract

**Background:**

Over the last century the incidence of cutaneous melanoma has increased worldwide, a trend that has also been observed in Brazil. The identified risk factors for melanoma include the pattern of sun exposure, family history, and certain phenotypic features. In addition, the incidence of melanoma might be influenced by ethnicity. Like many countries, Brazil has high immigration rates and consequently a heterogenous population. However, Brazil is unique among such countries in that the ethnic heterogeneity of its population is primarily attributable to admixture. This study aimed to evaluate the contribution of European ethnicity to the risk of cutaneous melanoma in Brazil.

**Methodology/Principal Findings:**

We carried out a hospital-based case-control study in the metropolitan area of Sao Paulo, Brazil. We evaluated 424 hospitalized patients (202 melanoma patients and 222 control patients) regarding phenotypic features, sun exposure, and number of grandparents born in Europe. Through multivariate logistic regression analysis, we found the following variables to be independently associated with melanoma: grandparents born in Europe—Spain (OR = 3.01, 95% CI: 1.03–8.77), Italy (OR = 3.47, 95% CI: 1.41–8.57), a Germanic/Slavic country (OR = 3.06, 95% CI: 1.05–8.93), or ≥2 European countries (OR = 2.82, 95% CI: 1.06–7.47); eye color—light brown (OR = 1.99, 95% CI: 1.14–3.84) and green/blue (OR = 4.62; 95% CI 2.22–9.58); pigmented lesion removal (OR = 3.78; 95% CI: 2.21–6.49); no lifetime sunscreen use (OR = 3.08; 95% CI: 1.03–9.22); and lifetime severe sunburn (OR = 1.81; 95% CI: 1.03–3.19).

**Conclusions:**

Our results indicate that European ancestry is a risk factor for cutaneous melanoma. Such risk appears to be related not only to skin type, eye color, and tanning capacity but also to others specific characteristics of European populations introduced in the New World by European immigrants.

## Introduction

Populations on different continents vary considerably in their predisposition to disease, probably as a result of adaptation to local selective factors such as climate and available nutrients but also as a result of genetic ancestry.

The population of Brazil is a diverse mix of ethnic groups. After Brazil was discovered in 1500, Portuguese settlers and (later) African slaves intermarried with the indigenous peoples of Brazil. In the 19th and 20th centuries, there were waves of immigration to Brazil from various European countries. Between 1872 and 1975, Brazil received more than 5.4 million immigrants [1]: 34% from Italy; 29% from Portugal; 14% from Spain; 5% from Japan; 4% from Germany; 2% from Syria or Lebanon; and 12% from other countries. Because of extensive admixture among these various groups, the Brazilian population shows great variability in terms of skin pigmentation, phenotypic features, and genomic structure [2].

Cutaneous melanoma is associated with high mortality rates. Worldwide, its incidence has increased progressively over the last century, as have the levels of exposure to ultraviolet (UV) radiation, a well-known risk factor, and it has been suggested that certain environmental and genetic factors also increase melanoma risk [3]. Recent increases in the incidence of melanoma have also been observed in Brazil [4]. These increases are probably related to changes in individual habits, including the pursuit of recreational activities in the sun [4].

The identified risk factors for melanoma include the pattern of sun exposure [5], a family history of melanoma, a high number of nevi, and certain phenotypic features, such as skin type, hair color, and eye color [6], [7]. Some studies have suggested that ethnicity influences the occurrence of melanomas. The European phenotype, characterized by blue or green eyes, together with light-colored hair, pale skin, and low tanning ability, has consistently been associated with an increased risk of malignant melanoma [7], [8]. It seems reasonable that the increased risk associated with European ethnicity should be attributed to lighter skin pigmentation. However, European ethnicity as predictor of melanoma independent of skin pigmentation has been poorly investigated. Most studies of melanoma have been carried out in Europe, North America, or Australia, mainly involving White populations in geographic regions where there is little admixture. Ethnicity has been investigated in only a few studies and for limited approaches [9]–[13].

It seems logical that admixture between European immigrants and indigenous peoples would have increased susceptibility to melanoma in the population of Brazil.

The aim of this case-control study was to assess the importance of ethnicity as a risk factor for melanoma in an admixed population. Our main hypothesis was that European ethnicity is predictor for cutaneous melanoma independent of phenotype.

## Methods

### Participants

Patients with melanoma were recruited from among those diagnosed between October 2004 and July 2008 at one of three facilities in the city of Sao Paulo, Brazil: *Hospital das Clínicas da Faculdade de Medicina da Universidade de São Paulo*; *Hospital do Câncer Antônio Cândido Camargo*; and *Instituto Brasileiro de Controle do Câncer*. The inclusion criteria were having received histopathological confirmation of cutaneous melanoma, being White, and being a resident of the metropolitan area of Sao Paulo. Patients were recruited during the pre-treatment phase. Age and sex frequency-matched controls were recruited from among cancer-free White patients hospitalized on the orthopedic or gastroenterology ward of the *Hospital das Clínicas*, who were confirmed as being melanoma-free upon recruitment. Controls also had to be residents of the metropolitan area of Sao Paulo. In both groups, only patients between 15 and 75 years of age were included.

The study was designed to detect a 1.9-fold increase in risk with a type I error of 0.05 and a type II error of 0.2 (80% statistical power). Assuming a 20% exposure rate in the control population and a 1∶1 control to case ratio, the minimum sample size was determined to be 420 (210 cases and 210 controls).

### Investigations Undertaken

During hospitalization, interviews were conducted by one of the three trained interviewers. Using a standardized questionnaire, we collected data related to sociodemographic characteristics (sex, age, income, and level of education); self-reported characteristics, including natural eye/hair color at 15 years of age, Fitzpatrick sun-reactive skin type [14], also known as skin phototype; and the history of UV exposure (use of tanning beds, use of sunscreen from birth to 9 years, number of sunburns during childhood/adolescence, and lifetime history of severe sunburn).

Participants were asked to classify the distribution of freckles on their face in childhood as either sparse or dense. Skin phototypes were defined as follows [14]: type I (always burns, never tans); type II (often burns, tans minimally); type III (rarely burns, tans well); and type IV (never burns, always tans). Cases and controls were asked if they had previously had moles or other pigmented lesions removed. The parental history of cancer was classified as negative if neither the mother nor the father had had cancer, and as positive if either or both parents had had cancer. The family history of skin cancer was classified as positive if one or more brothers, sisters, sons, or daughters had had any type of skin cancer.

Sunscreen use was categorized as follows: often; occasionally; or modified (e.g., changed from “never” in infancy or adolescence to “often” in adult life or from “often”/“occasionally” to “never”). Severe sunburn was defined as event of sun exposure resulting in skin redness and discomfort for at least two days. The frequency of sunburn in infancy and adolescence was classified as less than once/year, one to six times/year, or more than seven times/year.

On the basis of the American Joint Committee on Cancer T-category criteria and Breslow thickness [15], the case group tumors were categorized, by diameter, as follows: ≤1.0 mm (T1); 1.01–2.00 mm (T2); 2.01–4.00 mm (T3); and >4.0 mm (T4). We also classified the tumors by Clark level of invasion [16]: level I - lesions involving only the epidermis (in situ melanoma); level II - invasion of the papillary dermis; level III - invasion filling and expanding the papillary dermis; level IV - invasion into the reticular dermis; level V -invasion through the reticular dermis into the subcutaneous tissue.

Ethnic background was determined by the origin of the grandparents and was assessed by four approaches in terms of the frequency of migration to Brazil. Individuals with four Brazilian grandparents were used as the reference for all comparisons of ethnicity.

In the first approach, we considered the following European countries as a set: Spain, France, Italy, Portugal, Germany, Austria, Hungary, Yugoslavia, Lithuania, Poland, Romania, Russia, Switzerland, and Czechoslovakia. In this approach, cases and controls were distributed according to the total number of grandparents born in any of those countries.

In the second approach, we considered a smaller subgroup of European countries, including only Spain, France, Italy and Portugal. In this approach, cases and controls were also distributed according to the total number of grandparents born in any of these countries.

In the third approach, we considered a different subgroup, including Germany, Austria, Hungary, Yugoslavia, Lithuania, Poland, Romania, Russia, Switzerland, and Czechoslovakia. In this approach, cases and controls were also distributed according to the total number of grandparents born in any of these countries.

In the fourth approach, the countries with the highest rates of migration to Brazil were analyzed separately, whereas those with lower rates of migration to Brazil were grouped. Cases and controls were distributed as follows: those having one or more grandparents born in Spain (category 1); those having one or more grandparents born in Italy (category 2); those having one or more grandparents born in Portugal (category 3); those having one or more grandparents born in a Germanic or Slavic country (category 4); and those having one or more grandparents born in one European country and one or more grandparents born in a different European country (category 5).

### Ethics

The study protocol was approved by the Research Ethics Committee of all three facilities (*Hospital das Clínicas da Faculdade de Medicina da Universidade de São Paulo*; *Hospital do Câncer Antônio Cândido Camargo*; and *Instituto Brasileiro de Controle do Câncer*). All participating patients gave written informed consent. Participants under eighteen years old also had written informed consent from parents or legal guardians.

### Statistical Methods

To estimate the risk of melanoma associated with selected factors, we calculated odds ratios (ORs) and 95% confidence intervals (95% CIs) using unconditional logistic regression analysis. Because cases and controls were frequency-matched according to age and sex, all ORs were adjusted for age and sex. Level of education was also included, in order to control for socioeconomic status. Statistical significance was assessed using the likelihood ratio test. For ordered categorical variables, we employed tests for linear trend, categorizing the exposure variables and entering the scores as continuous values. Variables with a *p*<0.20 after adjustment for age, sex, and level of education were included in the multivariate models and were selected by backward stepwise regression. All four approaches to determining ethnicity presented a *p*<0.20. However, to avoid collinearity, only the fourth approach (because it was the most detailed approach) was included in multivariate analysis.

The STATA statistical program, version 10 (Stata Corp., College Station, TX, USA), was used for statistical analysis. All tests were two-tailed, and the level of statistical significance was set at *p*≤0.05.

## Results

A total of 212 patients with cutaneous melanoma were admitted to the three hospitals during the study period. Of those 212 melanoma patients, 6 (2.8%) declined to participate in the study and 4 (1.9%) died before the questionnaire was applied. The remaining 202 patients were included in the case group. We identified 230 eligible inpatient controls among inpatients on the orthopedics and gastroenterology wards. Of those 230 patients, 8 (3.5%) declined to participate in the study. Therefore, the control group comprised 222 patients. Proportion (%) of males in cases and controls were 49.5. The mean (SD) age in the case and control groups was 51.7 (14.3) years and 48.2 (14.8) years, respectively (Table 1).

**Table 1:** Socio-demographic characteristics of participants.

Risk factors	Cases* (%)	Controls* (%)
**Sex**		
Male	100 (49.5)	110 (49.5)
Female	102 (50.5)	112 (50.5)
**Age**		
15–29	12 (5.9)	25 (11.3)
30–39	28 (13.9)	35 (15.8)
40–49	51 (25.3)	58 (26.1)
50–59	51 (25.3)	50 (22.5)
60–69	35 (17.3)	30 (13.5)
70–79	25 (12.4)	24 (10.8)
**Age Average (SD)**	51.7 (14.3)	48.2 (14.8)
**Educational level**		
Until incomplete elementary	40 (19.2)	32 (14.9)
Elementary/Junior high school	24 (11.9)	57 (26.6)
Senior high school	48 (23.8)	78 (36.4)
College	89 (44.9)	47 (22.0)
**Income (Real** ** **)**		
Less than 720	23 (11.4)	43 (20.4)
721 a 1,200	25 (12.4)	37 (17.5)
1,201 a 2,400	32 (15.9)	48 (22.7)
Above 2,401	121 (60.2)	83 (39.3)

* Totals may vary because of missing value.

** One real is approximately U.S. dollar 0.59.

On the basis of the American Joint Committee on Cancer T-category criteria and Breslow thickness [15], we categorized the tumors as T1 in 61% of the cases, T2 in 16%, T3 in 14%, and T4 in 9%. When we classified the tumors by Clark level [16], 5% of the cases were level I (in situ), 23% level II, 49% level III, 20% level IV, and 3% level V.

Table 2 shows the distribution of cases and controls by hair/eye color, density of childhood freckling, skin phototype, history of pigmented lesion removal, and family history of skin cancer. Risk factors such as eye color (blue, green, or light brown), hair color (blond, red, or light brown), density of childhood freckling, skin phototype, and history of pigmented lesion removal were associated with an overall increase in melanoma risk. Blond/red hair color and blue/green eye color were associated with a 4-fold increase in the risk of melanoma. Family history of skin cancer or parental history of any cancer were not associated with melanoma risk.

**Table 2:** Odds ratios of melanoma associated with phenotypic features and family history of cancer.

Risk factor	Cases^a^ (%)	Controls^a^ (%)	OR (95% CI)^b^
**Hair color**			
Black or dark brown	60 (29.9)	114 (51.8)	1
Light brown	98 (48.8)	89 (40.5)	2.11 (1.36–3.26)
Blond or red	43 (21.4)	17 (7.7)	4.49 (2.32–8.70)
*p trend*			<0.0001
**Eye color**			
Black or dark brown	47 (23.5)	113 (51.1)	1
Light brown	100 (50.0)	79 (35.8)	2.90 (1.82–4.60)
Blue or green	53 (26.5)	29 (13.1)	4.51 (2.50–8.15)
*p trend*			<0.0001
**Childhood freckle distribution**			
Sparse	123 (62.1)	175 (81.0)	1
Dense	75 (37.9)	41 (19.0)	2.40 (1.52–3.81)
**Skin phototype** c			
IV	22 (10.9)	58 (28.0)	1
III	60 (29.7)	68 (32.9)	1.95 (1.05–3.63)
II	97 (48.0)	60 (29.0)	4.13 (2.27–7.51)
I	23 (11.4)	21 (10.1)	2.42 (1.10–5.29)
*p trend*			0.001
**History of pigmented lesion removal**			
No	86 (42.8)	179 (81.4)	1
Yes	115 (57.2)	41 (18.6)	5.38 (3.42–8.48)
**Parental history of cancer**			
No	121 (62.7)	122 (65.9)	1
Yes	72 (37.3)	63 (34.1)	1.02 (0.66–1.58)
**Family history of skin cancer** d			
No	191 (94.6)	208 (97.7)	1
Yes	11 (5.4)	5 (2.3)	1.93 (0.65–5.73)

a Totals may vary because of missing values.

b Adjusted for age, sex and educational level by unconditional logistic regression analysis. Statistical significance: likelihood ratio test.

c I: always burns, never tans; II: often burns, tans minimally; III: rarely burns, tans well; IV: never burns, always tans.

d Brothers, sisters, sons, and daughters.

As can be seen in Table 3, neither tanning bed use nor the use of sunscreen from birth to 9 years age was associated with occurrence of melanoma. A frequency of sunburn in childhood and adolescence was associated with a 4- to 8-fold increase in the risk of melanoma. A lifetime history of severe sunburn was also strongly associated with melanoma risk.

**Table 3:** Exposure to UV radiation, use of sunscreen, and history of sunburn.

Risk factor	Cases^a^ (%)	Controls^a^ (%)	OR (95% CI)^b^
**Use of tanning bed**			
No	185 (93.4)	201 (93.1)	1
Yes	13 (6.6)	15 (6.9)	1.03 (0.62–2.14)
**Use of sunscreen at 0–9 years of age**			
Often	8 (4.1)	18 (8.8)	1
Occasionally	2 (1.0)	3 (1.5)	0.99 (0.13–7.58)
Never/almost never	185 (94.9)	184 (89.8)	1.69 (0.67–4.25)
**Lifetime sunscreen use**			
Often	9 (4.5)	27 (12.7)	1
Occasionally	9 (4.5)	7 (3.3)	4.28 (1.12–16.39)
Never/almost never	85 (42.3)	101 (47.6)	2.02 (0.86–4.77)
Modified	98 (48.8)	77 (36.3)	2.62 (1.11–6.16)
**Sunburn at 0–14 years of age**			
<1 per year	72 (35.6)	126 (60.0)	1
1–6 per year	102 (50.5)	75 (35.7)	2.83 (1.78–4.50)
≥7 per year	28 (13.9)	9 (4.3)	6.22 (2.72–14.24)
*p trend*			<0.0001
**Sunburn at 15–19 years of age**			
<1 per year	55 (28.4)	126 (60.9)	1
1–6 per year	85 (43.8)	62 (30.0)	3.99 (2.35–6.79)
≥7 per year	54 (27.8)	19 (9.2)	8.48 (4.35–16.54)
*p trend*			<0.0001
**Severe sunburn (lifetime)**			
No	50 (24.8)	101 (48.3)	1
Yes	152 (75.2)	108 (51.7)	3.11 (1.99–4.87)

a Totals may vary because of missing values.

b Adjusted for age, sex and educational level by unconditional logistic regression analysis. Statistical significance: likelihood ratio test.

Table 4 presents the ethnicity of the sample, based on the four approaches taken. The first approach (analyzing 14 European countries as a set) and second approach (analyzing 4 southern European countries as a set) showed a trend, with a strong gradient, toward an association with the number of grandparents. Having 3 or 4 European grandparents was found to increase melanoma risk by approximately 4 times compared with having four Brazilian grandparents. When adjusting for phenotypic features (eye color, hair color, childhood freckle density, and skin phototype) the trend remained strong, indicating that the increased risk related to ethnicity is not solely attributable to specific physical features. The fourth approach produced a result similar to that of the first two approaches. For countries with higher rates of immigration to Brazil (Spain, Italy, and Germanic/Slavic countries), adjusting for phenotypic features reduced the magnitude of the risk but did not negate the statistical significance of this association. In the multivariate logistic regression (Table 5), the following variables were identified as independent predictors of melanoma: European ethnicity (Spanish, Italian, Germanic/Slavic, or mixed); eye color (green, blue, or light brown); history of pigmented lesion removal; lifetime history of no sunscreen use; sunburn in adolescence; and lifetime history of severe sunburn.

**Table 4:** Odds ratios of melanoma associated with European ethnicity.

Country of grandparent birth	Number of Grandparents	Cases^a^ (%)	Controls^a^ (%)	OR (95% CI)^b^	OR (95% CI)^c^
**Brazil (reference)**	Four	71 (35.9)	132 (62.0)	1	1
**European group 1** d					
	One	16 (8.1)	15 (7.0)	1.87 (0.87–4.00)	1.68 (0.74–3.81)
	Two	34 (17.2)	35 (16.4)	1.94 (1.31–3.23)	1.47 (0.78–2.77)
	Three or four	77 (38.9)	31 (14.5)	4.45 (2.53–7.82)	3.46 (1.87–6.41)
	*p trend*			<0.0001	<0.0001
**European group 2** e					
	One	12 (6.7)	11 (5.5)	1.43 (0.66–3.06)	1.46 (0.64–3.31)
	Two	30 (17.1)	31 (15.4)	2.03 (1.12–3.66)	1.51 (0.77–2.94)
	Three or four	62 (35.4)	27 (13.4)	4.24 (2.30–7.80)	3.52 (1.81–6.83)
	*p trend*			<0.0001	<0.0001
**European group 3** f					
	One	4 (4.2)	4 (2.8)	1.89 (0.45–7.92)	0.99 (0.19–5.15)
	Two	4 (4.2)	4 (2.8)	1.63 (0.38–7.07)	1.37 (0.30–6.20)
	Three or four	15 (16.0)	4 (2.8)	5.89 (1.82–19.08)	3.71 (0.94–14.61)
	*p trend*			0.01	0.07
**Spain** g	One or more	19 (9.6)	14 (6.6)	4.45 (1.78–11.14)	3.32 (1.24–8.92)
**Italy** g	One or more	46 (23.5)	29 (13.6)	4.62 (2.20–9.68)	3.34 (1.47–7.58)
**Portugal** g	One or more	18 (9.1)	17 (8.0)	3.01 (1.27–7.14)	2.65 (1.05–6.65)
**Germanic/Slavic subset** f **^,^** g	One or more	19 (9.6)	9 (4.2)	4.94 (1.94–12.57)	3.31 (1.15–9.48)
**≥2 European countries**	Two or more	24 (12.2)	12 (5.6)	4.63 (2.05–10.47)	3.44 (1.44–8.24)

a Totals may vary because of missing values.

b Adjusted for age, sex and educational level by unconditional logistic regression analysis. Statistical significance: likelihood ratio test.

c Adjusted for age, sex, level of education, eye color, hair color, freckle density, and skin phototype by unconditional logistic regression analysis. Statistical significance: likelihood ratio test.

d Spain, France, Italy, Portugal, Germany, Austria, Hungary, Yugoslavia, Lithuania, Poland, Romania, Russia, Switzerland, or Czechoslovakia.

e Spain, France, Italy, or Portugal.

f Germany, Austria, Hungary, Yugoslavia, Lithuania, Poland, Romania, Russia, Switzerland, or Czechoslovakia.

g Adjusted further for the number of European grandparents.

**Table 5:** Factors associated with melanoma in the multivariate logistic regression analysis.

Ethnicity	OR (95% CI)^a^
Brazilian	1
Spanish	3.01 (1.03–8.77)
Italian	3.47 (1.41–8.57)
Portuguese	2.32 (0.82–6.61)
Germanic or Slavic	3.06 (1.05–8.93)
Mixed European	2.82 (1.06–7.47)
**Eye color**	
Black or dark Brown	1
Light Brown	1.99 (1.14–3.84)
Blue or Green	4.62 (2.22–9.58)
*p trend*	<0.0001
**History of pigmented lesion removal**	
No	1
Yes	3.78 (2.21–6.49)
**Lifetime sunscreen use**	
Often	1
Occasionally	3.53 (0.59–21.21)
Never/almost never	3.08 (1.03–9.22)
Modified	2.74 (0.93–8.12)
**Sunburn at 15–19 years of age**	
<1 per year	1
1–6 per year	3.47 (1.83–6.59)
≥7 per year	4.60 (2.22–9.50)
*p trend*	<0.0001
**Severe sunburn (lifetime)**	
No	1
Yes	1.81 (1.03–3.19)

a Adjusted for age, sex, education level and the other factors shown in the table.

## Discussion

Summarizing our findings, data suggest that European ethnicity is a risk factor for cutaneous melanoma, with a strong trend toward greater risk associated with the number of European grandparents. This increased risk might be related not only to skin phototype but also to genetic polymorphisms introduced into the New World by European immigrants.

In this case-control study, we also corroborated the findings of other authors in relation to the role that certain phenotypic features play in increasing susceptibility to melanoma in an admixed population [5]–[8].

The history of Brazil involved waves of immigration and minimal social stigma associated with interethnic marriage [2]. The heterogeneity of its population is therefore the product of five centuries of admixture between and among individuals native to Brazil and those of African, Portuguese, German, Italian, Spanish, Middle Eastern or Asian descent.

In the present study, we found that European ethnicity was associated with an increased risk of cutaneous melanoma in Brazil, as previously suggested in a relatively small study conducted in the southern region of the country [12]. Very little has been published regarding cutaneous melanoma in other admixed populations [13]. Acton et al., analyzing the population of the American state of Alabama, found that being of Northern European descent was associated with the risk of cutaneous melanoma, although being of Mediterranean descent was not [9]. Loria et al. studied a sample of individuals born in Argentina and found a higher risk of cutaneous melanoma among those with four European grandparents [10]. Bakos et al. found that being a member of an indigenous population in Brazil was protective against melanoma and that being of European descent was an independent factor for the occurrence of melanoma [12]. To our knowledge, there have been no studies conducting a detailed investigation of European ethnicity in relation to melanoma, as we have done here.

Certain phenotypic features, such as light-colored hair, pale skin, and light eye color, are specific to Europe. The European population shows small genetic distances and few differences in population structure when compared with populations on other continents, suggesting a relatively homogenous continental population [17]. It seems reasonable that the increased risk associated with European ethnicity should be attributed to skin phototype [6]–[8]. However, European ethnicity persisted as an independent risk factor for melanoma, even after adjustment for phenotypic features. Phenotypic features are genetically determined by a relatively small number of genes [2] that were evolutionarily selected by environment, especially the amount of UV exposure [18]. The melanoma risk associated with European ethnicity might be related to factors that have yet to be extensively researched. This association might be explained by the influence of combinations of genetic polymorphisms related to ancestry rather than by phenotypic features. Various studies have found positive associations between genetic polymorphisms and the risk of cutaneous melanoma [19]–[23].

Studies carried out in other regions of the world have reported that certain phenotypic features increase the risk of melanoma [6], [7], [24]. In fact, there is strong evidence that melanoma development is associated with the UV index and latitude [24], [25]. In addition, a study conducted in the city of Sao Paulo during the 2005–2008 period showed that the UV index ranged from very high to extreme (according to the World Health Organization classification) on 65% of the days in the summer and in 40% of the days in the winter [27]. Therefore, Sao Paulo residents are continually exposed to high-intensity solar radiation and high UV indices, critical factors for the development of skin cancers, including melanoma [27].

We found that individuals with a history of sunburn were at an increased risk of developing melanoma. This was probably related to skin phototype. Individuals with skin phototype I or II produce less melanin, especially eumelanin, which acts as a physical barrier, scattering the incident UV light, and as a filter to reduce its penetration into the epidermis [28], [29]. Multiple episodes of sunburn can damage the genetic material, causing mutations that accumulate throughout life and promote the process of skin carcinogenesis. Recent studies indicate that increased sensitivity to sun exposure is also associated with polymorphisms and mutations in genes involved in skin pigmentation [30], [31].

All epidemiology studies are subject to limitations. Population controls, though preferable for reasons of validity, present more information bias than that affecting cases. Response of individuals selected from the general population tends to be worse than that from other types of controls because they are often less cooperative. We chose hospital-controls because they are more suitable when hospital cases are studied [32]. A case-control design is susceptible to confounders if there is differential ascertainment of risk factors between cases and controls. We minimized this aspect by standardizing our methods of data collection. In addition, the interviewers were blinded to the status (case or control) of the subjects. Non-differential misclassification could have occurred if subjects not truly having melanoma were included as cases, independent of their exposure status. This could result in an underestimation of the risk of cutaneous melanoma. To avoid this problem, we included only cases in which there was histopathological confirmation of the diagnosis. In case-control studies such as ours, there is a potential for recall bias. It is unlikely that ancestry was misclassified, because the birthplace of grandparents is relatively objective information. To address potential problems with recall for others self-reported variables, we enrolled only untreated cases, so that their responses were less likely to be influenced by changes that occurred after diagnosis. Therefore the associations with skin phototype, hair color, and history of pigmented lesion removal were comparable to those reported in previous studies. Nevertheless, some residual confounding cannot be completely ruled out. No association was found between melanoma risk and tanning bed exposure, a variable for which the occurrence of recall bias is more likely. Therefore, the degree of tanning bed exposure might may have been underreported [33]. Additionally caution is advised due to a relatively small sample that can result in lower precision with larger confidence interval.

Our results open new perspectives for research into the etiology of melanoma. The population of Brazil differs from those of other countries with high rates of immigration because of the admixture of various ethnicities. Therefore, we cannot assume that European ethnicity plays a similar role in the etiology of melanoma in other regions of the world. However, to determine whether the role of European ethnicity in other admixed populations is similar to that observed for Brazil, it would be useful to conduct new studies in countries such as the United States, Australia and other Latin America countries. In addition, the identification of genetic polymorphisms specific to a given ethnicity could form the basis for investigations of molecular markers of cutaneous melanoma. Such studies should consider the diversity of European ethnicity
